# Evolutionary Dynamics and Age-Dependent Pathogenesis of Sub-Genotype VI.2.1.1.2.2 PPMV-1 in Pigeons

**DOI:** 10.3390/v12040433

**Published:** 2020-04-11

**Authors:** Peng Xie, Libin Chen, Yifan Zhang, Qiuyan Lin, Chan Ding, Ming Liao, Chenggang Xu, Bin Xiang, Tao Ren

**Affiliations:** 1 College of Veterinary Medicine, South China Agricultural University, Guangzhou 510642, China; 34gnep@163.com (P.X.); chenlibin777@foxmail.com (L.C.); Hopeyifan@126.com (Y.Z.); linqiuyan05@163.com (Q.L.); mliao@scau.edu.cn (M.L.); chgangxu@126.com (C.X.); 2 Key Laboratory of Animal Vaccine Development, Ministry of Agriculture, Guangzhou 510642, China; 3 National and Regional Joint Engineering Laboratory for Medicament of Zoonosis Prevention and Control, Guangzhou 510642, China; 4 Key Laboratory of Zoonosis Prevention and Control of Guangdong Province, Guangzhou 510642, China; 5 Shanghai Veterinary Research Institute (SHVRI), Chinese Academy of Agricultural Sciences (CAAS), Shanghai 200241, China; shoveldeen@shvri.ac.cn

**Keywords:** PPMV-1, sub-genotype VI.2.1.1.2.2, phylogenetic tree, age, pigeon, pathogenesis, transmission

## Abstract

Pigeon paramyxovirus type 1 (PPMV-1) infection causes high morbidity in pigeons, resulting in a significant burden to the poultry industry. In this study, we isolated three PPMV-1 strains from diseased pigeons collected in Guangdong Province, South China, from June 2017 to April 2019. Genetic analysis revealed that these three PPMV-1 strains and most of the PPMV-1 strains isolated from China after 2011 were clustered into sub-genotype VI.2.1.1.2.2. Our Bayesian analysis revealed that the VI.2.1.1.2.2 viruses might have originated in Europe. Phylogeographic analyses revealed that East and South China might have played a key role in seeding the VI.2.1.1.2.2 PPMV-1 epidemic in China. To characterize the effect of age at infection on the outcome of PPMV-1 infection in pigeons, we investigated the pathogenesis and transmission of the pigeon/Guangdong/GZ08/2017 (GZ08) virus in 3-, 6-, and 12-week-old pigeons. Two of six 12-week-old pigeons inoculated with GZ08 survived, and all of the 3- and 6-week-pigeons inoculated with GZ08 died. Moreover, the GZ08 virus could be transmitted to 3-, 6-, and 12-week-old naïve contact pigeons. The lethality of the GZ08 virus through contact with 3-, 6-, and 12-week-old pigeons was 100%, 66.7%, and 0%, respectively, suggesting that the transmissibility of the GZ08 virus was stronger in young pigeons. These findings demonstrated that East and South China was the epicenter for dissemination of VI.2.1.1.2.2 PPMV-1, and age at infection has an impact on the outcome of PPMV-1 infection in pigeons.

## 1. Introduction

Newcastle disease (ND) is a worldwide infectious disease that causes significant economic losses to the poultry industry. Its causative agent is the virulent Newcastle disease virus (NDV), a member of the genus *Orthoavulavirus* of the family *Paramyxoviridae* and the subfamily *Avulavirinae*. The NDV genome is a single-stranded, negative-sense RNA, coding six structural proteins, including nucleoprotein, phosphoprotein, matrix protein, fusion (F) protein, hemagglutinin–neuraminidase and large protein, and two non-structure proteins, V and W [1]. According to the updated unified classification system of NDVs based on the complete F gene sequence (1–1662 nt) proposed by Dimitrov et al. [2], NDV can be grouped into two major groups, class I and class II, with a single serotype. Until now, 20 genotypes in class II and only one genotype in class I have been identified. All but two virulent NDVs belong to class II [3].

Genotype VI NDVs, also referred to as pigeon paramyxovirus type 1 (PPMV-1) viruses, are variant strains of NDV associated with infections of Columbiformes, including wild and domestic pigeons and doves. Since PPMV-1 was first identified in the Middle East, particularly in Iraq, in 1978 [4], they have been detected in many other countries, including the USA [5], Great Britain, and Russia [6], and are known to have caused the third epizootic during the 1980s [7]. Moreover, PPMV-1 is also considered to be responsible for ND outbreaks in chickens and could increase their virulence in chickens after several passages in chickens [8,9]. Phylogenetic analysis revealed that PPMV-1 strains showed genetic diversity, with at least 15 sub-genotypes [3,10,11,12]. According to the updated classification criteria of NDV sub-genotypes (in which the cutoff value of the nucleotide distance was increased from 3% to 5%), all PPMV-1 strains were divided into VI.1, VI.2.1, VI.2.1.2, VI.2.2.1, VI.2.2.2, VI.2.1.1.1, VI.2.1.1.2.1, and VI.2.1.1.2.2 [2].

Although most PPMV-1 strains are nonpathogenic to chickens, they can cause high morbidity and mortality in pigeons, leading to a degree of economic loss. The primary clinical signs in pigeons after being infected with PPMV-1 are neurological signs, such as paralysis, torticollis, and a twisted neck and head [13,14]. The disease induced by PPMV-1 in pigeons was first confirmed in China in 1985 [15]. Pigeons are often sold in live poultry markets in China due to the dietary preference of Chinese people, and live poultry trading might drive the dissemination of PPMV-1. Currently, PPMV-1 circulates in a majority of provinces in China [15,16,17]. A large number of clinical cases found that younger pigeons have significantly higher morbidity and mortality than older pigeons, indicating that age may have an impact on the pathogenicity of PPMV-1 in pigeons [13,18]. In our previous studies, PPMV-1 induced obvious clinical signs, but not death, in 15-week-old pigeons [19]. However, some research showed that the mortality of the PPMV-1-infected pigeons was 50% in 1-month-old pigeons [20], 70% in over 2-month-old pigeons [21], and 80% in 4-week-old pigeons [13]. These findings indicated that the pathogenicity of PPMV-1 in pigeons may be closely related to age.

In the current study, we isolated three PPMV-1 strains from diseased pigeons collected in Guangdong Province, from June 2017 to April 2019, and investigated the prevalence, molecular evolution, and phylogeography of these viruses. Moreover, we also characterized the effect of age at infection on the outcome of PPMV-1 infection in pigeons and analyzed the antigenic variation between epidemical PPMV-1 in South China and the commercial vaccine LaSota strain.

## 2. Methods and Materials

### 2.1. Ethics Statement

Animals were treated humanely and with regard for alleviation of suffering. All animal experiments were approved by the South China Agricultural University Experimental Animal Welfare Ethics Committee (permit number: 2019-V021; permit date: 16 March 2019).

### 2.2. Virus Isolation and Identification

The PPMV-1 strains were isolated from diseased pigeons suspected of having PPMV-1 infection in Guangdong Province, South China, from June 2017 to April 2019. Respiratory and neurological signs, including gasping and twisting of the head and neck, were observed in these diseased pigeons. The collected tissue samples were homogenized in 1.0 mL of phosphate-buffered saline (PBS) supplemented with antibiotics (penicillin, 2000 U/mL; amphotericin B, 2000 U/mL; streptomycin, 2 mg/mL) using TissueLyser II (Qiagen, Hilden, Germany). After centrifugation at 5000× *g* for 10 min, the supernatants were propagated in 10-day-old specific-pathogen-free (SPF) chicken embryos. The presence of NDVs was confirmed via hemagglutination (HA), HA-inhibition (HI) assay, and reverse transcription polymerase chain reaction (RT-PCR), as described previously [22]. After three rounds of plaque-purification on primary chicken embryo fibroblast cells, these viruses were amplified in SPF chicken embryos.

### 2.3. Sequence Analysis

The virus genome was isolated from infective allantoic fluid using RNeasy Mini Kit (Qiagen) and transcribed into cDNA with M-MLV (Moloney Murine Leukemia Virus) Reverse Transcriptase (RNase H-; TaKaRa, Dalian, China). PCR amplification was performed using 12 pairs of overlapping primers specific to the complete genomes of NDV, as described previously [23]. To determine the 3′- and 5′-ends of the viral genomes, rapid amplification of cDNA end (RACE) was carried out using SMARTer™ RACE cDNA, as reported previously [24]. The PCR products were cloned into pMD19-T and sequenced at Sangon Biotechnology (Guangzhou, China).

### 2.4. Genetic and Phylogenetic Analyses

A total of 364 complete F gene sequences of PPMV-1 submitted before 31 December 2019, were collected from GenBank. All sequences were aligned by the version 7.058 program. A maximum likelihood [25] tree was reconstructed based on the complete F gene (1–1662 nt) sequence using the IQ-TREE software [26], implemented by the GTR + F + I + G4 model with 1000 bootstrap replicates, using the genotype II NDV strain LaSota (GenBank: AF077761) as an outgroup.

In order to determine the temporal structure of all the PPMV-1 strains in the VI.2.1.1.2.2, a regression of root-to-tip genetic distance was performed for these sequences using the TempEst software [27] based on the unrooted ML tree of VI.2.1.1.2.2 viruses. The Bayesian Markov chain Monte Carlo (MCMC) method was selected to infer the divergence time of sub-genotype VI.2.1.1.2.2 PPMV-1 strains in BEAST version 1.10.4 [28]. The TN93 + G4 substitution model was selected using ModelFinder with Bayesian information criteria [29]. To compare models of molecular clocks (the strict molecular clock model and the uncorrected lognormal relaxed molecular clock) and demographic change (constant size, exponential growth, and Bayesian skyline), the log-marginal likelihood was estimated by path sampling (PS) and stepping-stone (SS) sampling [30]. All analyses were run 100 million steps with a sampling frequency every 10,000 steps, and a burn-in of 10% was discarded. Convergence of all parameters (i.e., effective sample sizes >200) was confirmed visually using Tracer version 1.7 [31]. The maximum clade credibility (MCC) tree was inferred through the TreeAnnotator (part of BEAST version 1.10.4) and visualized via FigTree version 1.4.2. A similar statistical method was performed to estimate the most recent common ancestor (TMRCA) for all the groups in sub-genotype VI and group I and II in sub-genotype VI.2.1.1.2.2. To investigate the demographic history of sub-genotype VI.2.1.1.2.2 PPMV-1 strains, we used a coalescent-based Bayesian skyline plot, and a piecewise-constant skyline model was implemented using the BEAST version 1.10.4 software.

### 2.5. Bayesian Phylogeographic Analysis

To understand the spatial diffusion patterns of VI.2.1.1.2.2 PPMV-1 strains, the ancestral geographical regions, diffusion rates, and migration patterns of these viruses were analyzed by the asymmetric continuous-time Markov chain phylogeographic model with the Bayesian Stochastic Search Variable Selection (BSSVS) in the BEAST version 1.10.4 software [32]. Due to only one sample data being collected exclusively from each of India and Egypt, which may increase the uncertainty of the ancestral reconstruction, we removed these sequences. China can be divided into six geographical regions, including East, North, Northeast, Northwest, South, Central, and Southwest, based on the traditional regions. The geographical regions of China, in addition to Europe, were selected and coded as discrete states using a strict clock model and Bayesian Skyline coalescent. The MCMC was run for 100 million steps, with sampling every 10,000 steps. Then, the significant nonzero transition rates were identified using Bayes factors (BFs) in SPREAD3 version 0.9.7 [33]. To confirm the reliability of analysis, the BSSVS ran three times independently. Significant migration pathways were determined on the basis of the combination of both BF ≥ 3 and a mean indicator of 0.5. BF ≥ 1000 indicated decisive support, 100 ≤ BF < 1000 indicated very strong support, 10 ≤ BF < 100 indicated strong support, and 3 ≤ BF < 10 indicated statistically significant support. To assess the reliability of the most plausible location at the root node, we compared the results with those from 10 replicate datasets in which the location states were randomized among the sequences.

### 2.6. Pathogenicity and Transmission in Pigeons of Different Ages

Three-week-old pigeons were purchased from a commercial pigeon farm (Guangzhou, China) and housed in isolators under negative pressure with food and water provided ad libitum. None of the pigeons had any history of disease or vaccination against NDV. Prior to animal experimentation, all pigeons were confirmed to be seronegative for NDV antibodies by the HI (Hemagglutination Inhibition) test with GZ08 antigens and negative for NDV in oropharyngeal and cloacal swabs by RT-PCR, as described previously [22].

Twelve 3-week-old pigeons were inoculated intranasally with 10^6^ egg infectious dose (EID_50_) of the GZ08 virus in a 200-μL volume. Eight hours after inoculation, three 3-week-old pigeons, inoculated with 200 μL PBS via the same route, were cohoused with these inoculated pigeons as the naïve contact group. The pigeons in the control group (*n* = 10) were inoculated intranasally with 200 μL PBS. At 3 and 7 days post-infection (DPI), we euthanized three inoculated pigeons and determined the virus titers in the brain and lungs. To detect virus shedding, oropharyngeal and cloacal swabs were collected at 3, 5, 7, 9, 11, 13, 17, and 21 DPI and suspended in 1000 μL PBS with antibiotics (penicillin and amphotericin B, 4000 U/mL; streptomycin, 2 mg/mL). All collected samples were stored at −80 °C until further use. Virus titers were determined by EID_50_ assay, as described previously [23]. At 21 DPI, serum samples from all surviving birds were collected for serological testing by the HI test with GZ08 antigens.

Three-week-old pigeons were housed in isolators until they were 6 and 12 weeks old. The procedures conducted on 6- and 12-week-old pigeons followed the same design as that for 3-week-old pigeons.

### 2.7. Cross-Reactivity HI Assay

The cross-reactivity HI assay between the commonly used vaccine LaSota and the PPMV-1 strain, GZ08, was conducted as described previously [34]. Briefly, 3-week-old pigeons were intramuscularly injected with inactivated oil-emulsified LaSota or GZ08 virus. After 14 days, a booster immunization was performed. Serum samples from all pigeons were collected 7 days after the second immunization. All serum samples were inactivated at 56 °C for 0.5 h and stored at −80 °C. The HI titers of the GZ08 and LaSota antisera were determined by the HI test with four HA units of antigen (GZ08 and LaSota). The *R*-value was calculated using the formula from a previous report [35].

### 2.8. Statistical Analysis

Statistical analysis was performed using GraphPad Prism version 8.0.1 software (GraphPad Software Inc., San Diego, CA, USA). Differences of virus titers in 3-, 6-, and 12-week-old pigeons were compared by one-way analysis of variance (ANOVA). Compared to the 3-week-old pigeon group, *p* < 0.05 and *p* < 0.01 were considered to indicate statistically significant differences, unless stated otherwise.

## 3. Result

### 3.1. Virus Isolation and Identification

After passage in SPF chicken eggs, three PPMV-1 strains were identified by HA/HI and RT-PCR assays, designated as pigeon/Guangdong/GZ08/2017 (GZ08), pigeon/Guangdong/SZ12/2018, and pigeon/Guangdong/HY25/2019 under GenBank numbers MN893303–MN893305.

### 3.2. Phylogenetic Analysis

**Figure 1:**
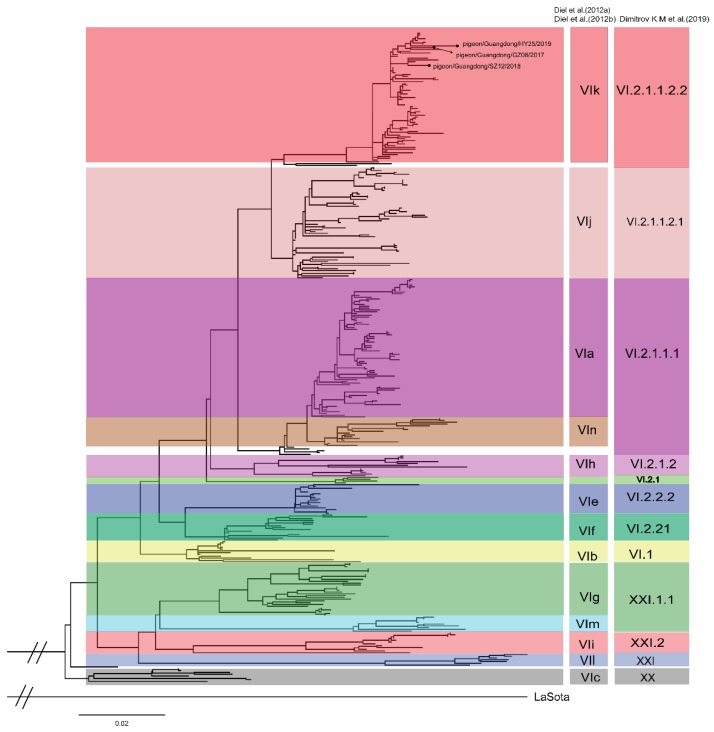
The maximum likelihood tree of PPMV-1 strains. The tree was reconstructed based on the complete F gene (1–1662 nt) sequence using the IQ-TREE software, implemented by the GTR + F + I + G4 model with 1000 bootstrap replicates, using the genotype II NDV strain LaSota as the outgroup.

**Figure 2:**
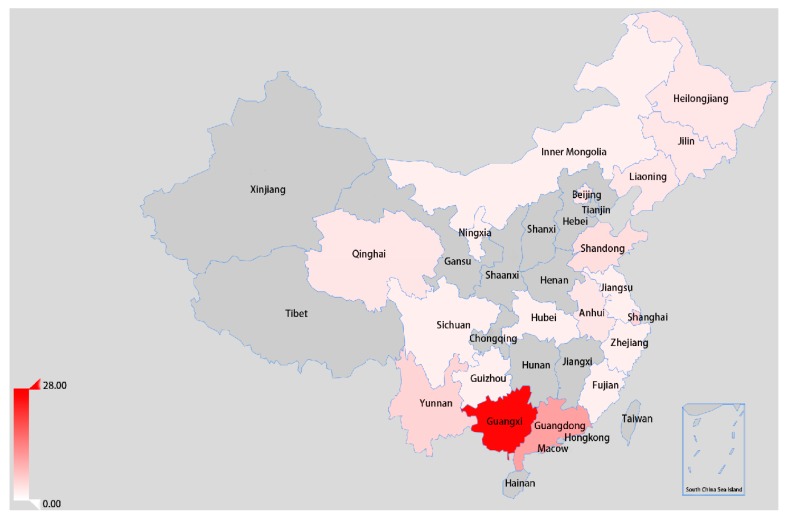
Distribution of VI.2.1.1.2.2 pigeon paramyxovirus type 1 (PPMV-1) isolated from China. The Chinese provinces where the presence of VI.2.1.1.2.2 PPMV-1 has been reported are indicated in red. All data were obtained from GenBank in NCBI [37].

Based on the classification system for assigning NDVs proposed by [36], an ML tree was constructed on the basis of the complete F gene (1–1662 nt) sequence. As shown in Figure 1, the three isolates identified in this study were grouped into sub-genotype VIk, as in a previous study [12]. The sub-genotype VIk PPMV-1 strains and the other two viruses (PPMV-1/Belgium/05-03936-8/2005 and NL/human/2003) were described as VI.2.1.1.2.2, according to the updated unified phylogenetic classification system [2]. A total of 75 VI.2.1.1.2.2 viruses were identified, including the three isolates in this study. Notably, most PPMV-1 strains isolated from China were identified as VI.2.1.1.2.2 after the year 2011. Moreover, these viruses were mainly isolated from South China, including Guangxi and Guangdong Province (Figure 2).

The root-to-tip regression analysis of the VI.2.1.1.2.2 viruses showed that the correlation coefficient and *R*^2^ were 0.8864 and 0.6175, respectively, confirming the presence of structure in the data. Next, the best model was estimated by comparing the marginal likelihood estimates, using PS and SS. The results demonstrated that the prior use of a strict clock and Bayesian skyline coalescent tree provided the best data fit (Table 1). The mean substitution rate estimated in our Bayesian analysis was 1.317 × 10^−3^ subs/site/year (95% credibility interval, 1.008 × 10^−3^–1.562 × 10^−3^). The rate estimated from the root-to-tip regression (1.46 × 10^−3^ subs/site/year) was also similar to the results of the Bayesian analysis.

**Figure 3:**
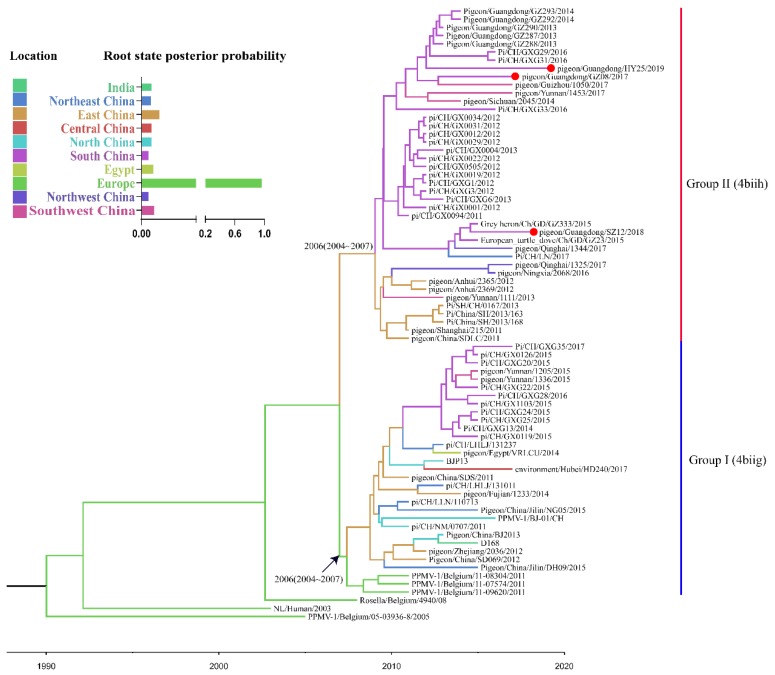
Maximum clade credibility tree of the complete F gene of the sub-genotype VI.2.1.1.2.2 PPMV-1 strains. The tree was constructed using the BEAST version 1.10.4 software. Branch colors denote inferred location states, with the key for colors displayed. The strains from this study are indicated in red solid circles. All VI.2.1.1.2.2 PPMV-1 strains contain 72 isolates available in GenBank as of 31 March 2019, and the three isolates identified in our study. The root state posterior probabilities for the eight regions are shown in the inset panel.

The time-scaled MCC tree of VI.2.1.1.2.2 PPMV-1 isolates based on the complete F gene showed that all VI.2.1.1.2.2 PPMV-1 viruses isolated from China were generally divided into groups I–II (Figure 3). Groups I and II were identified as 4biig and 4biih, respectively, in the recent study [38], based on a classification system described by Aldous et al. [4]. Group I included three isolates from Belgium, one isolate from India, one isolate from Egypt, and 26 isolates from China, with TMRCA in 2006 (95% highest posterior density (HPD), 2004–2007). Group II included 41 isolates from China, with TMRCA in 2006 (95% HPD, 2004–2007). We also ran the analysis using all sequences in genotype VI (Table S1). The Bayesian analysis placed the root of the tree in Europe, with a posterior probability of 0.96 (Figure 3). To assess the reliability of the most plausible location at the root node, we compared the results with those from 10 replicate datasets in which the location states were randomized among the sequences (Figure S1). The root state posterior probability of Europe was outside the range of probability (0.02–0.07) obtained in the analyses of the randomized datasets, indicating that the results were reliable.

**Figure 4:**
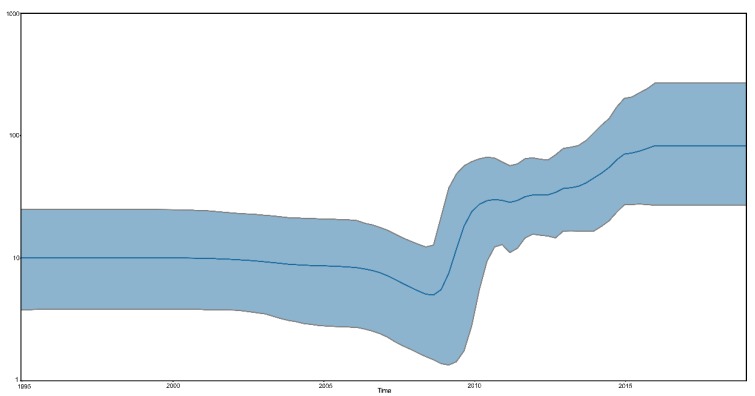
Bayesian skyline plot of the complete F gene of the sub-genotype VI.2.1.1.2.2 PPMV-1 strains. The dark blue line shows the mean value of genetic diversity, and the light blue shading shows the 95% confidence interval.

To illustrate the population size of sub-genotype VI.2.1.1.2.2 NDVs, the Bayesian skyline coalescent was reconstructed to reveal that the relative genetic diversity of sub-genotype VI.2.1.1.2.2 NDVs. As shown in Figure 4, the population size of the sub-genotype VI.2.1.1.2.2 PPMV-1 was relatively constant before 2008, then expanded between 2008 and 2016. After 2016, the sub-genotype VI.2.1.1.2.2 PPMV-1 population was also relatively constant.

### 3.3. Spatial Dynamics of the VI.2.1.1.2.2 PPMV-1 Strains

**Figure 5:**
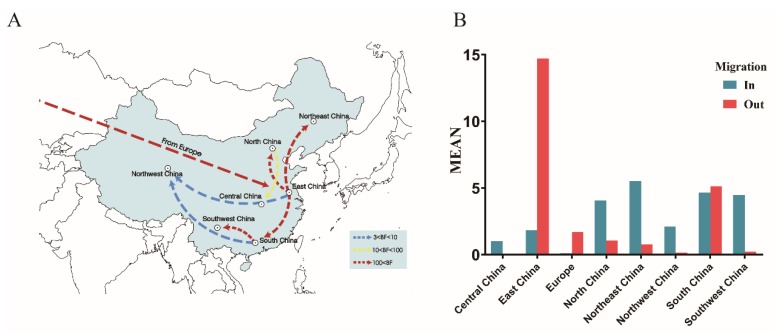
Spatial diffusion of VI.2.1.1.2.2 viruses. (**A**) Spatial diffusion pathways. (**B**) Histogram of the total number of state transitions.

To estimate the global circulation of the VI.2.1.1.2.2 PPMV-1 strains, eight geographic regions (including Europe, Central China, East China, North China, Northeast China, Northwest China, South China, and Southwest China) were divided according to geographical location, and a BSSVS procedure was implemented (Table 2 and Figure 5). Europe, the most probable root state, showed a statistically significant association with East China (BF = 336). The routes between East China and the other regions of China, including Northeast China (BF = 920), North China (BF = 320), South China (BF = 324), and Northwest China (BF = 7.7), were also identified. Furthermore, high BF values (BF ≥ 1000) between South China and Southwest China were identified. High mean rates were observed, indicating migration from East China to Northeast China, South China, and North China, and from South China to Southwest China. These results revealed that East and South China might have played a key role in seeding the VI.2.1.1.2.2 PPMV-1 epidemic in China. The number of observed state changes related to the migration from East and South China was much higher than from any other regions, which further confirmed this finding.

### 3.4. Pathogenicity of the GZ08 Virus in 3-, 6-, and 12-Week-Old Pigeons

**Figure 6:**
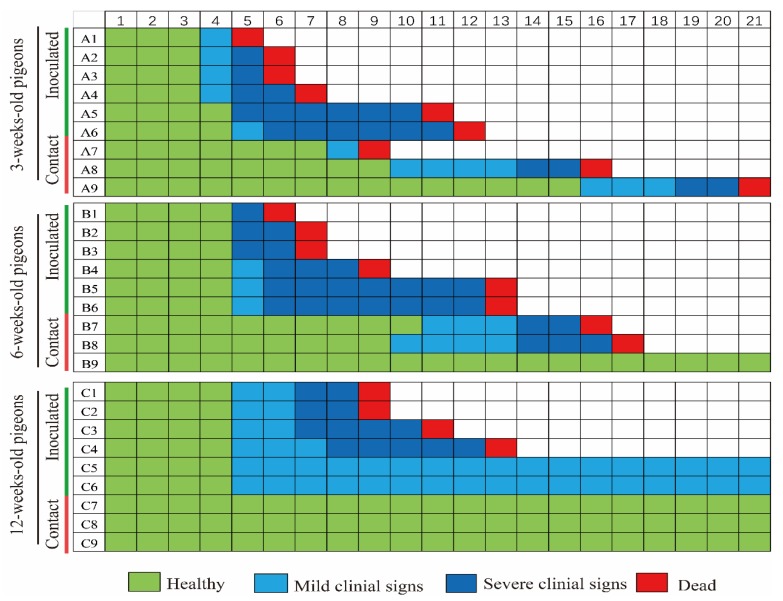
Clinical signs and lethality of the GZ08 virus in 3-, 6-, and 12-week-old pigeons. Clinical inspection was performed once daily, and symptoms were scored according to severity.

All of 3- and 6-week-old pigeons inoculated with GZ08 died within 12 and 13 DPI, respectively, whereas two of the six 12-week-old pigeons that were inoculated survived. In 3-week-old inoculated pigeons, mild clinical signs such as depression were observed at 4 DPI; one pigeon died, and four pigeons exhibited severe clinical signs, including twisting of the head and neck, incoordination, and paralysis, at 5 DPI (Figure 6). Clinical signs were first observed in 6-week-old pigeons at 5 DPI. In 12-week-old pigeons, mild clinical signs were first observed at 5 DPI, and a total of 4 out of 6 pigeons died during the experimentation period. The mean HI titer of the two surviving 12-week-old pigeons was 9.50 ± 0.71 log2 (2/2).

**Figure 7:**
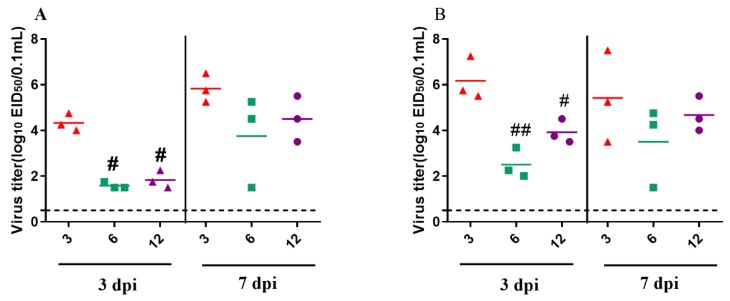
Virus titers in tissues of 3-, 6-, and 12-week-old pigeons inoculated with the GZ08 virus at 3 and 7 DPI in (**A**) brains and (**B**) lungs. Data are presented as means ± standard deviation (SD). The dashed black line denotes the lower limit of detection. Statistical analysis was performed using GraphPad Prism version 8.0.1. Differences in virus titers in 3-, 6-, and 12-week-old pigeons were compared by one-way ANOVA. Compared to the 3-week-old pigeon group, ^#^ *p* < 0.05 and ^##^ *p* < 0.01 were considered to indicate statistically significant differences, unless stated otherwise.

To determine the replication of the GZ08 virus in 3-, 6-, and 12-week-old pigeons, three inoculated pigeons were euthanized at 3 and 7 DPI. The virus was detectable in the brain and lungs of 3-, 6-, and 12-week-old pigeons (Figure 7). In 3-week-old inoculated pigeons, the mean virus titers at 3 DPI in the brain and lungs were 4.33 and 6.25 log10 EID_50_, respectively, and these values increased to 5.83 and 5.42 log10 EID_50_, respectively, at 7 DPI. In 6-week-old pigeons, the mean viral loads in the brain and lungs were 1.58 and 2.5 log10 EID_50_, respectively, at 3 DPI. In 12-week-old pigeons, the mean viral loads in the brain and lungs were 1.83 and 3.91, respectively, at 3 DPI, and 4.5 and 4.67 log10 EID_50_, respectively, at 7 DPI. Notably, the virus titers in the brain were significantly higher than those in 6- or 12-week-old infected pigeons (*p* < 0.05). At 7 DPI, virus titers of the tested tissues of all the inoculated pigeons, except the 3-week-old group, increased, compared with those at 3 DPI. Generally, virus titers were higher in 3-week-old pigeons than in 6- and 12-week-old pigeons for the investigated time periods.

To determine virus shedding in inoculated pigeons of different ages, oropharyngeal and cloacal swabs were collected from all chickens at 3, 5, 7, 9, 11, 13, 17, and 21 DPI. Shedding of the GZ08 virus could be detected in both oropharyngeal and cloacal swabs of inoculated 3-, 6-, and 12-week-old pigeons during the experimentation period (Table 3).

### 3.5. Transmission of PPMV-1 in 3-, 6-, and 12-Week-Old Pigeons

To determine the intraspecific horizontal transmission of PPMV-1, three pigeons served as the naïve contact group and were cohoused with inoculated pigeons in each group. The naïve contact 3-week-old pigeons began to show clinical signs at 8 DPI, and their mortality was 100% (Figure 6). However, no obvious clinical signs or death were observed in naïve contact 12-week-old pigeons throughout the experimentation period, but the seroconversion rate was 100%. The HI titer of the sole surviving pigeon in the contact 6-week-old group was 8 log2, and the mean HI titer of the three surviving contact 12-week-old pigeons was 7.67 ± 0.58 log2 (3/3). The mortality of GZ08 in-contact 3-, 6-, and 12-week-old pigeons was 100%, 66.67%, and 0%, respectively. Shedding of the GZ08 virus in the oropharyngeal and cloacal swabs of 3-, 6-, and 12-week-old pigeons was detectable during the experimentation period (Table 3). In summary, the GZ08 virus was transmissible in pigeons of different ages via direct contact, but the transmissibility was stronger in younger pigeons.

### 3.6. Cross-Reactivity HI Test

To analyze the antigenic variation of PPMV-1 and the commercial vaccine strain LaSota, a cross-reactive HI assay was performed. The *R* value was 0.597, indicating minor antigenic differences between the GZ08 strain and the commonly used vaccine strain LaSota.

## 4. Discussion

Since PPMV-1 was first identified in China in 1985 [15], PPMV-1 infection remains enzootic in pigeons despite vaccination, such as LaSota, leading to a degree of economic loss [17,38]. In this study, we isolated three PPMV-1 strains from diseased pigeons collected in Guangdong Province, South China, from June 2017 to April 2019. The ML tree based on the complete F gene demonstrated that these three viruses belonged to sub-genotype VIk, based on the classification described by Diel et al. [36], or to VI.2.1.1.2.2, based on the updated unified classification system proposed by Dimitrov et al. [2]. Notably, all VI.2.1.1.2.2 viruses, except two strains, PPMV-1/Belgium/05-03936-8/2005 and NL/Human/2003, were clustered into sub-genotype Vik, mainly because the cutoff value of nucleotide distance was increased from 3% to 5% in the updated unified classification system.

According to the MCC tree of the VI.2.1.1.2.2 PPMV-1, the viruses isolated from Europe were located at the root of the tree with the highest root state posterior probability of 0.96, indicating that Europe might have been the origin of VI.2.1.1.2.2 viruses. For the precise of the analysis, we excluded single data in India or Egypt. The deduction was further supported by the migration link from Europe to East China in the diffusion processes of VI.2.1.1.2.2 PPMV-1. Previous studies demonstrated that the European continent was the epicenter of global dissemination of PPMV-1 due to the movement of pigeons [39,40]. Cai et al. [16] also proved that the VI viruses (P4 and W4) of China originated in Europe. However, the crown group of VI.2.1.1.2.2 viruses was dated to the year 1989 (95% HPD, 1984–1994), more than a decade before those European strains were isolated. The lack of sequence during this period led to uncertainty that Europe was the source of the VI.2.1.1.2.2 viruses. Sampling bias is an important factor that should also be considered in models. Hicks and his coworkers used the tip swap analysis to assess the impact of sampling on the estimated viral diffusion patterns of avian paramyxovirus-1 and found that the sample was biased toward the presence of earlier sequences within the dataset [40]. In this study, we compared the Bayesian analysis results with those from 10 replicate datasets in which the location states were randomized among the sequences. The root state posterior probability of Europe was outside the range of probability obtained in the analyses of the randomized datasets, suggesting that these results were reliable. Firstly, the data sampling was unbalanced on the timeline, and only a few strains were isolated in earlier years, which may have been caused by ignorance to the disease and a lack of organized epidemiological investigations. Another important point is the sampling bias based on geography regions preference. The data scarcity may reflect that other regions were free of PPMV-1 sub-genotype, or just restricted sampling coverage, and data deficiency from other continents may have contributed to this bias. Similar causes may also have led to VI.2.1.1.2.2 PPMV-1 isolation enrichment in China, in which pigeons were reared as poultry, and a more frequent and progressive sampling could have been conducted; in European, pigeons under surveillance are mainly racing pigeons, which also led to sampling bias [9]. For the above-mentioned reasons, it could not be excluded that VI.2.1.1.2.2 PPMV-1 may have originated in other regions. Thus, based on the One Health Model, an epidemiological investigation of PPMV-1 should be conducted worldwide. Our phylogeographic analysis also revealed a general southeastern-to-central region dispersal of VI.2.1.1.2.2 PPMV-1 in China. Due to strict environment policy of the local government, the pigeon industry in China gradually shifted from East and South China to inter-mainland China in recent years, possibly providing a clue regarding virus diffusion.

In our Bayesian analysis, the estimated substitution rate of the F gene from VI.2.1.1.2.2 PPMV-1 viruses was 1.317 × 10^−3^ subs/site/year (95% credibility interval, 1.079 × 10^−3^–1.557 × 10^−3^), and similar rates were observed in Miller et al.’s [41] report for virulent NDVs, i.e., 1.32 × 10^−3^ (strict) and 1.70 × 10^−3^ (relaxed), and Ramey et al.’s [42] report, i.e., 1.12 × 10^−3^ (strict) and 1.32 × 10^−3^ (relaxed) for wild-bird origin class I NDVs, and 1.02 × 10^−3^ (strict) and 1.10 × 10^−3^ (relaxed) for class II NDVs. However, the substitution rate of VI.2.1.1.2.2 PPMV-1 viruses was shown to be faster than those of virulent genotype II (7.05 × 10^−5^) and IX (2.05 × 10^−5^) NDVs in a recent study [43]. Notably, to control ND in pigeons, NDV vaccines are widely used in pigeons. Thus, PPMV-1 in domestic pigeons suffered the selective pressure of vaccination. To avoid this potential bias, the wild-bird-origin VI.2.1.1.2.2 PPMV-1 viruses were more appropriate for estimating the substitution rate. However, the gene sequences of VI.2.1.1.2.2 PPMV-1 from wild birds in different regions remain largely unknown. Therefore, we should strengthen epidemiological surveillance of PPMV-1 in wild birds.

A previous study found that PPMV-1 virus led to a 100% mortality in 1-day-old chickens by intratracheal and intracerebral inoculation, whereas no death was observed in 14-day-old chickens after being infected via the same route [44]. However, a recent study showed that the pathogenicity and transmission of virulent NDVs had no significant differences in young and adult chickens [45]. In our study, we found that age at infection had an impact on the outcome of PPMV-1 infection in pigeons, which was generally consistent with clinical cases in which the morbidity and mortality of younger pigeons were significantly higher than those of older pigeons [13,18]. These results indicated that the age of birds might affect PPMV-1, but not other NDV infections. The GZ08 virus was transmissible in pigeons of different ages via direct contact, which was consistent with the findings of previous studies [14,46]. Notably, the transmissibility was stronger in younger pigeons than in older pigeons. To effectively control ND in young pigeons, we should pay more attention to the vaccination of egg-laying pigeons, confirming high maternal antibody levels in young pigeons. The pathogenicity of NDV in birds correlated with the viral titers in tissues [46,47]. The PPMV-1 strain GZ08 could replicate in the brain and lungs of pigeons of different ages, but the virus titers of 3-week-old pigeons were significantly higher than those of 12-week-old pigeons. Thus, age could possibly influence the replication of VI.2.1.1.2.2 PPMV-1 in pigeons, thereby affecting the progression of the disease. Notably, there was no significant difference in viral titers between the ages of 6 and 12 weeks at 3 and 7 DPI. Besides virus replication, the virulence of NDV is also dependent on the host immune response [48,49,50]. Moreover, the development of the immune system is complete after hatching with exposure to foreign antigens. A previous study also demonstrated that the virulence of PPMV-1 is closely related to the inflammatory response in pigeons [19]. Further investigation of age-related immune response in pigeons should be conducted.

The cross-reactivity HI assay showed that the *R* value between the GZ08 strain and the commonly used vaccine LaSota was 0.59, indicating a minor antigenic difference between LaSota and GZ08. The results were similar to those of previous research [24,34]. A large number of reports confirmed that antigen matching between vaccine and epidemic strains could increase the effectiveness of vaccines against epidemic strains [51,52,53,54]. The presence of outbreaks caused by PPMV-1 also suggested that commonly used vaccines like LaSota are insufficient to control the disease, and virus neutralization assay and challenge studies are necessary to further confirm the efficacy of the LaSota vaccine to protect against PPMV-1 [55]. Therefore, an antigenically matched vaccine against PPMV-1 is critically needed to control ND in pigeons.

In conclusion, we isolated three VI.2.1.1.2.2 PPMV-1 from Guangdong Province, from June 2017 to April 2019, and demonstrated that East and South China was the epicenter for dissemination of VI.2.1.1.2.2 PPMV-1. We also showed that the age at infection has an impact on the outcome of PPMV-1 infection in pigeons. To control PPMV-1 infection in pigeons, it is necessary to develop antigenically matched vaccines and to strengthen the biosecurity measures that prevent viruses from infecting pigeons.

## Figures and Tables

**Table 1:** Candidate phylodynamic models for VI.2.1.1.2.2 PPMV-1 strains.

Model of Rate Variation	Coalescent Tree Prior	Log Marginal Likelihood (PS)	Log Marginal Likelihood (SS)	TMRCA (Year)	Substitution Rate (Subs/Site/Year)
Strict clock	Bayesian skyline	−5796.79	−5796.03	1989 (1984–1993)	1.317 × 10^−3^ (1.079 × 10^−3^–1.557 × 10^−3^)
Strict clock	Constant Size	5803.62	5802.99	1987 (1983–1992)	1.279 × 10^−3^ (1.047 × 10^−3^–1.516 × 10^−3^)
Strict clock	Exponential Growth	−5800.19	−5800.65	1989 (1984–1993)	1.309 × 10^−3^ (1.071 × 10^−3^–1.553 × 10^−3^)
Uncorrelated lognormal relaxed clock	Bayesian skyline	−5800.38	−5799.56	1989 (1982–1996)	1.352 × 10^−3^ (1.062 × 10^−3^–1.667 × 10^−3^)
Uncorrelated lognormal relaxed clock	Constant Size	−5809.58	−5808.59	1985 (1975–1993)	1.239 × 10^−3^ (9.598 × 10^−4^–1.515 × 10^−3^)
Uncorrelated lognormal relaxed clock	Exponential Growth	−5807.17	−5805.57	1991 (1984–1999)	1.355 × 10^−3^ (1.045 × 10^−3^–1.657 × 10^−3^)

TMRCA: the most recent common ancestor.

**Table 2:** Statistically supported migration rates of VI.2.1.1.2.2 PPMV-1 estimated from the F gene.

From	To	Mean Migration Rate	BF ^a^	Indicator ^b^
South China	Northwest China	0.580	3.460	0.355
East China	Northwest China	1.005	7.714	0.551
North China	Central China	0.828	33.919	0.844
East China	North China	3.900	320.421	0.981
East China	South China	4.441	324.242	0.981
Europe	East China	1.654	336.261	0.982
East China	Northeast China	5.140	920.264	0.993
South China	Southwest China	4.224	14,123.503	1.000

^a^ Bayes factor (BF) > 100 indicates decisive support for migration between locations. Only statistically supported migrations with BF > 3 are shown. ^b^ Posterior probability of observing a nonzero migration rate in the sampled trees.

**Table 3:** Virus shedding of the GZ08 virus from oropharyngeal and cloacal swabs in 3-, 6-, and 12-week-old pigeons.

		Days Post-Inoculation (Number Positive/Total)															
Group		3		5		7		9		11		13		17		21	
		OP	CL	OP	CL	OP	CL	OP	CL	OP	CL	OP	CL	OP	CL	OP	CL
3 weeks	Infection ^a^	6/12	10/12	4/9	7/9	3/6	5/6	1/3	1/3	1/3	2/3	-	-	-	-	-	-
	Contact ^b^	0/3	1/3	0/3	1/3	0/3	1/3	1/2	2/2	2/2	2/2	1/2	2/2	1/1	1/1	-	-
6 weeks	Infection	3/12	10/12	6/9	9/9	3/8	7/8	2/4	4/4	2/4	4/4	1/2	1/2	-	-	-	-
	Contact	0/3	1/3	0/3	1/3	0/3	1/3	1/3	2/3	1/3	3/3	1/3	3/3	1/2	1/2	0/1	0/1
12 weeks	Infection	9/12	11/12	8/9	8/9	4/9	8/9	1/6	4/6	1/4	4/4	1/3	2/3	0/2	1/2	0/2	0/2
	Contact	0/3	1/3	0/3	1/3	1/3	1/3	1/3	1/3	2/3	3/3	1/3	3/3	0/3	2/3	0/3	1/3

OP: oropharyngeal swabs; CL: cloacal swabs; -: all pigeons died at the end of the observation. ^a^ Pigeons were inoculated with the GZ08 virus. ^b^ Naïve contact chickens cohoused with those inoculated.

## Supplementary Materials

The following are available online at https://www.mdpi.com/1999-4915/12/4/433/s1, Figure S1: Results of the location-randomization analysis, Table S1: The TMRCA of different sub-genotypes in genotype VI.
