# Supplementary material for: Evolutionary Dynamics and Age-Dependent Pathogenesis of Sub-Genotype VI.2.1.1.2.2 PPMV-1 in Pigeons
Source: Viruses. 2020 Apr 11;12(4):433. doi: 10.3390/v12040433 (PMC7232354; doi:10.3390/v12040433)
Supplement: Supplementary file 1 [file viruses-12-00433-s001.pdf]

Table S1. The TMRCA of different sub-genotypes in genotype VI.

Sub-Genotype	TMRCA	95%HPD
VI.1	1964	1959–1968
VI.2.1	1975	1971–1978
VI.2.1.1.1	1991	1990–1993
VI.2.1.1.2.1	1993	1991–1995
VI.2.1.1.2.2	1987	1985–1990
VI.2.1.2	1975	1971–1978
VI.2.2.1	1972	1968–1976
VI.2.2.2	1972	1968–1976
VI.2.1.1.2.2-Group I (4biig)	2006	2004–2007
VI.2.1.1.2.2-Group II (4biih)	2006	2004–2007

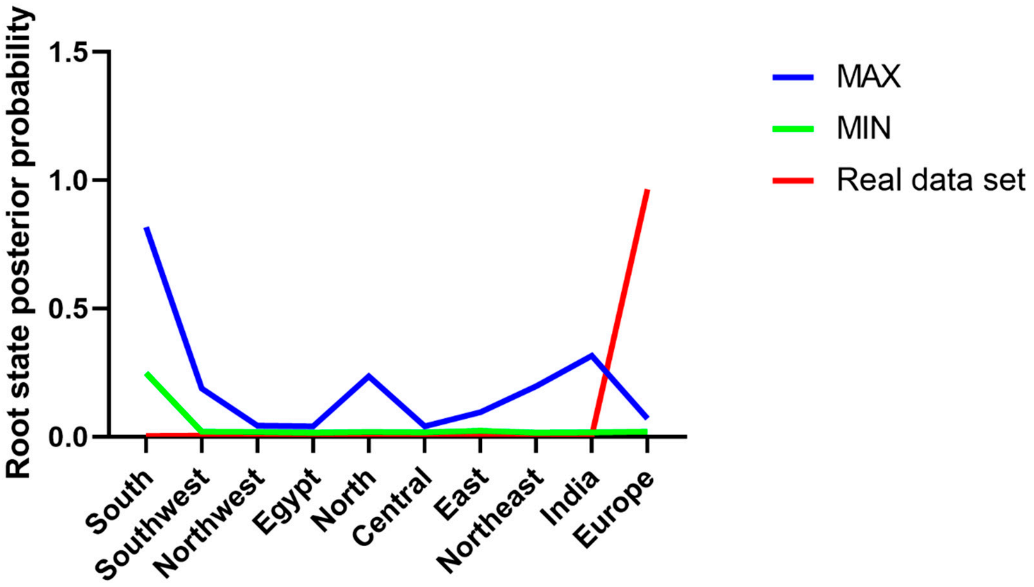

Figure S1. Results of the location-randomization analysis. Posterior probabilities of root locations are indicated in the y-axis. The maximum and minimum values for the root state posterior probability estimated from 10 replicate subsamples inferred from 10 location-randomized replicates are shown by blue and green lines, respectively. The values for the root state posterior probability estimated from the original data set are shown by the red line.
